# Conventional and neuropsychological criteria for mild cognitive impairment show similar prognostic value for dementia across 12 years in a non-clinical setting

**DOI:** 10.1038/s41598-025-04275-y

**Published:** 2025-06-05

**Authors:** Simone Galati, Michele Rossi, Federica Del Signore, Giulia Locatelli, Antonio Guaita, Elena Rolandi

**Affiliations:** 1 Golgi Cenci Foundation, Corso San Martino 10, 20081 Abbiategrasso (MI), Italy; 2 Department of Brain and Behavioral Sciences, University of Pavia, Pavia, Italy; 3 Department of Psychology, University of Milan-Bicocca, Milan, Italy

**Keywords:** MCI diagnosis, Neuropsychological criteria, Conventional criteria, Stability, Progression to dementia, Subjective Memory Impairment, Dementia, Diagnosis

## Abstract

Making early and informative diagnoses of mild cognitive impairment (MCI) is highly important for planning timely and appropriate interventions aimed at dementia risk reduction. However, there is currently no agreement on the MCI criteria, leading to wide heterogeneity in the prognosis of MCI patients and high reversion rates. Our study aimed to compare the prognostic value of Conventional (Petersen/Winblad) and Neuropsychological (Jak/Bondi) criteria for the diagnosis of MCI. We directly compared the ability of each classification method to predict progression to dementia and the stability of the diagnosis over 12 years in a population-based sample of 1021 older adults without dementia. The relative impact of subjective complaints and objective impairment on clinical progression was further evaluated. Baseline MCI diagnosis with the Neuropsychological and Conventional criteria was associated with a comparable risk of dementia over time. Across the study period, the Neuropsychological criteria led to more consistent diagnoses (63.2% vs. 43.2%). The copresence of subjective memory complaints and objective impairment at baseline was associated with increased dementia risk within both diagnostic frameworks. These results further support the use of comprehensive neuropsychological assessment to make timely and appropriate MCI diagnoses and show the added prognostic value of subjective complaints.

## Introduction

Mild cognitive impairment (MCI) is considered an intermediate stage between normal cognitive aging and early dementia and is characterized by objective cognitive impairment and preserved daily functioning^1–6^.

The formulation of an early MCI diagnosis is highly important for planning timely and appropriate interventions aimed at dementia risk reduction. However, to date, no definite consensus exists on how to diagnose MCI, with wide heterogeneity across different settings in the instruments used, the thresholds adopted and the number of tests required to define cognitive impairment^7,8^. This has led to substantial variation in dementia risk estimates, with annual conversion rates ranging from less than 5–20%^8^. At the same time, individuals with MCI could revert to normal cognition, with an estimate ranging between 14.4 and 55.6%^9–11^.

Clinical MCI diagnosis is usually based on the criteria for mild neurocognitive disorder in the Diagnostic and Statistical Manual of Mental Disorders, which require evidence of modest cognitive decline from a previous level of performance in one or more cognitive domains and preserved independence in everyday activities^12^. However, no clear recommendation is provided regarding the level of cognitive impairment required for the diagnosis, and a standardized neuropsychological assessment, although preferable, is not mandatory. Conversely, two main approaches to MCI diagnosis are adopted in the research setting based on comprehensive neuropsychological assessment.

In 1999, Petersen and colleagues developed criteria for the diagnosis of amnestic MCI that focused on subjective memory complaint, memory impairment, preserved general cognitive functions, normal daily activity and absence of dementia^3^. These criteria were revised in 2004 by the International Working Group to include impairment in other cognitive domains and to distinguish between the amnestic and nonamnestic forms of MCI^4,13–15^. These new recommendations, also called “Conventional criteria”, determine the presence of MCI when all of these criteria are met: self- or informant-reported cognitive complaint, objective impairment in one or more cognitive domains (at least one cognitive test scoring 1.5 standard deviations below the mean of the normative sample), preserved or minimally impaired daily functioning, and absence of dementia^5,15^. In 2009, Jak and colleagues proposed the Neuropsychological criteria, which differ from the Conventional criteria in that they do not require subjective cognitive complaints but require two impaired test scores within the same cognitive domain, with a cutoff score of 1 standard deviation below the mean of the normative sample^7^. These criteria for objective cognitive impairment help achieve a balance between sensitivity and reliability in the diagnosis of MCI^7,16^. In summary, both Conventional and Neuropsychological criteria require comprehensive neuropsychological evaluation but differ in the threshold established to define cognitive impairment, in the number of impaired tests needed, and in the consideration of subjective memory complaints to make a diagnosis of MCI.

Studies that have directly compared the performance of Conventional and Neuropsychological criteria on clinically relevant outcomes, such as the conversion rate and diagnostic stability, have shown contrasting results. Some researchers have shown that considering only one test below the cutoff to diagnose MCI results in incorrect classification of MCI subtypes, higher false-positive rates and higher reversion rates^6,7,16–19^. Individuals diagnosed with MCI according to the Neuropsychological criteria tend to remain more stable or progress to having dementia compared with those diagnosed according to the Conventional criteria^6,16,19^.

On the other hand, the Neuropsychological criteria require more tests for MCI classification, which are not always obtainable, and a recent well-designed study reported similar reversion rates between the Conventional and the Neuropsychological criteria^5^. In addition, this study reported that considering the presence of subjective complaints when the Neuropsychological criteria were adopted led to a lower reversion rate (48.3% versus 54.7%). Another study further demonstrated similar levels of conversion to dementia for both criteria^19^. On the other hand, other studies have shown that the requirement of subjective complaints for MCI diagnosis with Conventional criteria could lead to a misdiagnosis when the patient is unaware and tends to underestimate his or her difficulties^20,21^. Moreover, several studies have shown that the presence of subjective cognitive decline in the absence of objective cognitive impairment could be the earliest sign of dementia and Alzheimer’s disease^22,23^.

Thus, further studies are needed to clarify which diagnostic approach is more suitable for making a stable and informative MCI diagnosis, with a higher probability of clinical progression.

The research described above has several limitations that our study aimed to overcome. First, these studies were conducted mainly in America or Northern Europe with conventional samples; thus, further studies in different settings and with increased sample representativeness are needed. Second, few studies in the field have assessed the specific contribution of subjective memory complaints with or without objective cognitive impairment. Furthermore, previous studies had variable durations of follow-up (from 17 months to 9.7 years), with only three of them following participants for more than 5 years^5,6,19^.

Therefore, the overarching aim of the present study was to compare the prognostic value of Conventional and Neuropsychological criteria for MCI diagnosis in a population-based sample of older adults aged between 70 and 74 years of age at baseline and during 12 years of follow-up with periodic multidimensional assessment. Specifically, we empirically compared the ability of each set of criteria to detect individuals who subsequently progressed to dementia within the study period and the diagnostic consistency over time. Second, we further assessed the relative contributions of subjective memory complaints and objective cognitive impairment to clinical progression within different diagnostic frameworks.

## Results

### Study sample

**Fig. 1:**
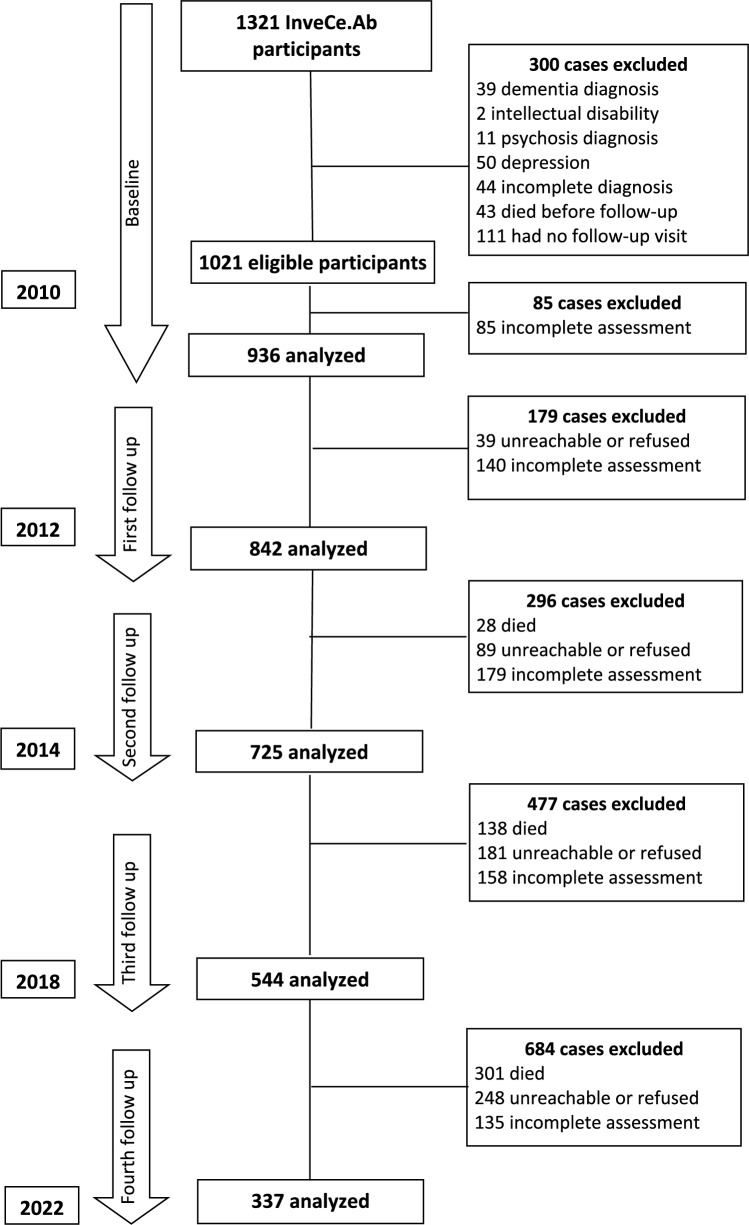
Flow chart of case selection.

Figure 1 shows the flow chart of case selection for the present study at each assessment wave. At baseline, 50 patients were excluded for depression, 11 for psychosis, two for intellectual disability, 39 for a baseline dementia diagnosis and 44 for not completing the neuropsychological assessment. Furthermore, 43 patients died before follow-up, and 111 did not attend any follow-up visit, resulting in a final sample of 1021 eligible individuals. Excluding those with incomplete neuropsychological assessments, we conducted our analysis on a sample of 936 individuals at baseline, of which 479 (52.1%) were female, with an average age of 72.1 years (SD = 1.3) and an average education level of 7.2 years (SD = 3.3). Among these individuals, 173 were diagnosed with MCI based on the Conventional criteria (18.5%), whereas 410 were diagnosed with MCI based on the Neuropsychological criteria (43.8%). The diagnostic outcomes at each time point according to the Conventional and Neuropsychological criteria are reported in Supplementary Tables S1 and S2, respectively.

The consistencies of MCI diagnosis significantly differed (McNemar’s test = 179.1, p value < 0.001). The two sets of criteria demonstrated minimal agreement in MCI diagnosis (Kappa index = 0.279) and low similarity (Jaccard index = 0.304).

### Prediction of dementia

**Fig. 2:**
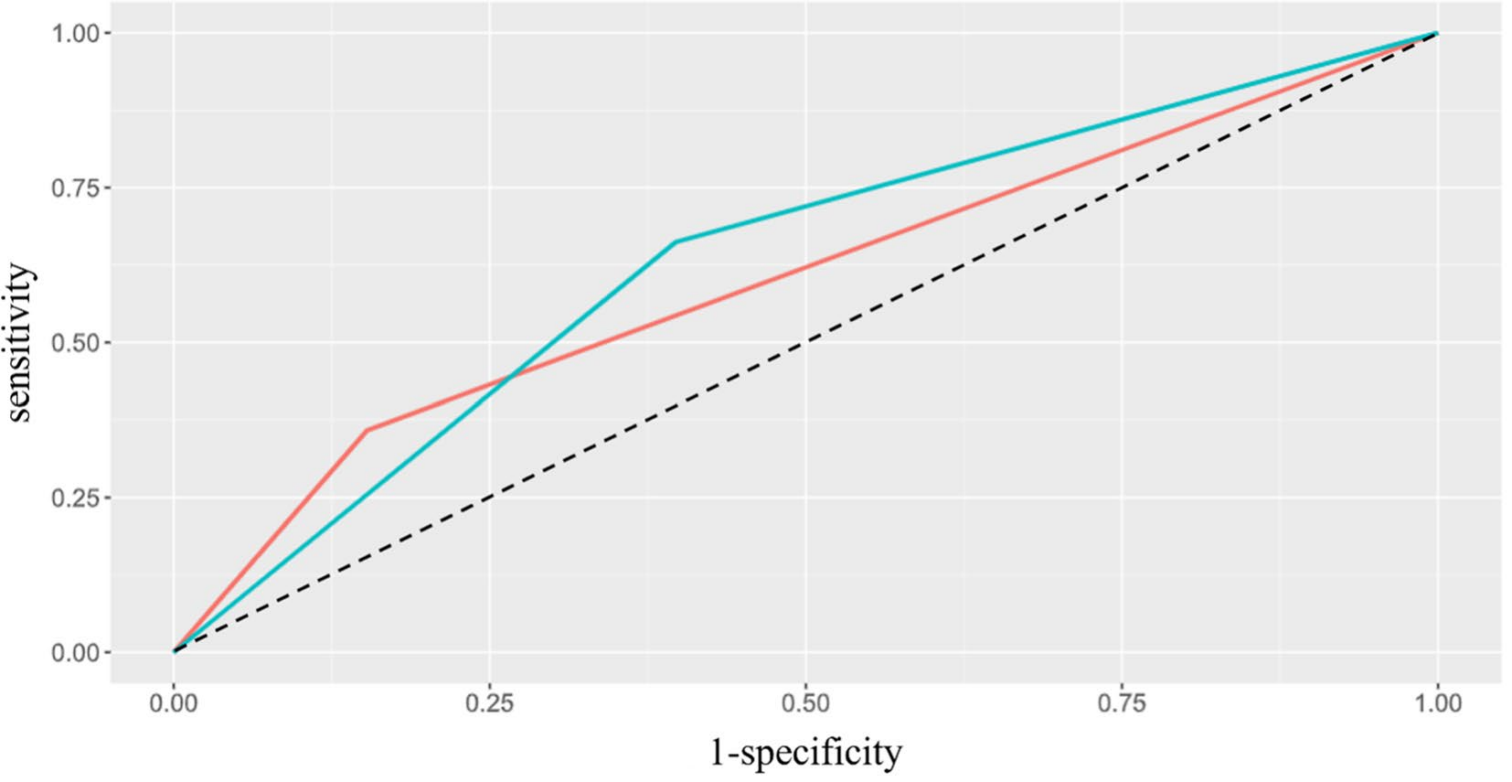
In the plot we see the comparison of the ROC curves relating to the classification of incident dementia by the two criteria. (Dashed: Random classifier, red: Conventional criteria, green: Neuropsychological criteria).

During the 12-year observation period, 15.5% (n = 145) of the participants developed dementia. Table 1 and Fig. 2 illustrate the sensitivity and specificity analysis using ROC curves, revealing no statistically significant difference between the operational characteristics of the two sets of criteria at baseline in classifying incident dementia over the following 12 years. In particular, baseline MCI diagnosis using the Conventional criteria yielded a sensitivity of 35.9% and a specificity of 84.7%, corresponding to a positive predictive value (PPV) of 30.1% (n = 52 patients with incident dementia) and a negative predictive value (NPV) of 87.8%. When the Neuropsychological criteria were used, baseline MCI diagnosis displayed a sensitivity of 66.2% and a specificity of 60.3%, corresponding to a PPV of 23.4% (n = 96 patients with incident dementia) and an NPV of 90.7%. The comparison of the Cox proportional hazards models adjusted for age, sex, and education, shown in Table 2, indicates a comparable risk of developing dementia within the next 12 years among individuals diagnosed with MCI using either the Neuropsychological criteria or Conventional criteria. When both criteria were concurrently considered (Model 3), the HRs were slightly attenuated but remained statistically significant for both criteria.

**Table 1:** Comparison of the areas under the ROC curves (AUC) of the two criteria using the DeLong test.

Variable	AUC	AUC lower	AUC upper	Z	p-value
Conventional criteria	0.603	0.562	0.644	49.00	< 0.001
Neuropsychological criteria	0.633	0.590	0.675	61.52	< 0.001
Difference	−0.030	−0.078	0.018	−12.20	0.223

**Table 2:** Comparison of Cox models assessing the predictive validity for dementia onset of baseline MCI diagnosis based on conventional (Model 1) and neuropsychological criteria (Model 2), plus a combined model concurrently considering the two classifications (Model 3).

	Model 1	Model 2	Model 3
MCI with conventional criteria	2.84 (0.18)***		2.11 (0.19)**
MCI with neuropsychological criteria		2. 72 (0.18)***	2.18 (0.19)***
AIC	1746	1743	1730
R^2^	0.044	0.046	0.061
Num. events	145	145	145
Num. obs	936	936	639
PH test	0.452	0.023	0.047

Hazard ratio (SD), model goodness-of-fit indices (AIC and R^2^), number of events and observations, and proportional hazards test (PH test) are reported. The reference group is cognitively normal subjects.

***p < 0.001; **p < 0.01; *p < 0.05.

### Diagnostic consistency

Consistent MCI diagnosis over 12 years was more common (McNemar’s test = 32.9, p value < 0.001) when using the Neuropsychological criteria (n = 177, 63.2%) compared with the Conventional criteria (n = 121, 43.2%) in the subsample of subjects with a non-uncertain diagnosis of MCI when using both criteria (n = 280). However, there was minimal agreement (Kappa index = 0.366) and low similarity (Jaccard index = 0.528) in diagnostic consistency between the two sets of criteria.

Table 3 shows the comparisons of the main characteristics of the sample according to diagnostic consistency categories within each diagnostic framework. For both diagnostic classifications, the cognitively normal participants were slightly younger, while education and sex were comparable. The incidence of dementia was lower among individuals with normal cognition and reverters than in those with consistent or uncertain diagnoses, with the latter having a more rapid conversion for both criteria. Considering study participation, the number of evaluations was greater in reverters, whereas individuals with an uncertain diagnosis were later diagnosed using both criteria. The comparison of Cox proportional hazards models adjusted for age, sex, and education (Table 4) to assess the effect of diagnostic consistency on dementia incidence revealed that consistent MCI diagnoses using the Neuropsychological criteria were associated with a slightly greater risk of developing dementia than when using the Conventional criteria. For both criteria, reverters presented a risk comparable to that of cognitively normal participants. Individuals with an uncertain MCI diagnosis displayed an increased risk of dementia when using both criteria, with similar numbers of assessments. Furthermore, when the Neuropsychological criteria were used, individuals with an uncertain MCI diagnosis presented the highest risk of dementia, even compared with those with consistent MCI diagnosis. When the two classification methods were included in the same model (Model 3), all HRs were slightly attenuated but remained significant.

**Table 3:** Descriptive statistics of the sample according to MCI diagnostic consistency considering conventional or neuropsychological criteria.

	Conventional criteria consistency				
	Cognitively normal	Reverters	Consistent	Uncertain	p-value
	n = 488	n = 192	n = 133	n = 145	
Age at baseline	71.97 (1.25)^c^	72.18 (1.23)^bc^	72.31 (1.29)^a^	72.17 (1.33)^ab^	0.019
Female	238 (48.8%)	100 (52.1%)	75 (56.4%)	81 (55.9%)	0.277
Education	7.00 (3.17)	7.66 (3.76)	7.11 (3.03)	6.74 (3.22)	0.054
Incident dementia	47 (9.6%)^a^	20 (10.4%)^a^	43 (32.3%)^b^	38 (26.2%)^b^	< 0.001
Time to dementia	90.09 (40.56)^ab^	108.40 (32.44)^a^	109.63 (40.21)^a^	77.92 (46.09)^b^	0.004
Number of Assessment	4.00 [2.00, 5.00]^b^	5.00 [2.00, 5.00]^a^	4.00 [2.00, 5.00]^b^	4.00 [2.00, 5.00]^b^	< 0.001
Wave at first MCI diagnosis	/	2.00 [1.00, 4.00]^b^	2.00 [1.00, 4.00]^b^	4.00 [1.00, 5.00]^a^	< 0.001
Number of MCI diagnosis	/	2.00 [1.00, 4.00]^b^	2.00 [2.00, 5.00]^a^	1.00 [1.00, 1.00]^c^	< 0.001

	Neuropsychological criteria consistency				
	Cognitively normal	Reverters	Consistent	Uncertain	p-value
	n = 232	n = 246	n = 285	n = 195	
Age at baseline	71.91 (1.19)^c^	71.99 (1.24)^bc^	72.28 (1.32)^a^	72.13 (1.28)^ab^	0.006
Female	130 (56.0%)	117 (47.6%)	139 (48.8%)	108 (55.4%)	0.141
Education	7.19 (3.29)	7.24 (3.40)	7.14 (3.09)	6.80 (3.46)	0.524
Incident dementia	15 (6.5%)^a^	9 (3.7%)^a^	73 (25.6%)^b^	51 (26.2%)^b^	< 0.001
Time to dementia	119.07 (31.18)^a^	106.33 (38.49)^ab^	103.84 (38.49)^a^	73.61 (45.27)^b^	< 0.001
Number of assessment	4.00 [2.00, 5.00]^b^	5.00 [2.00, 5.00]^a^	4.00 [2.00, 5.00]^b^	4.00 [2.00, 5.00]^b^	< 0.001
Wave at first MCI diagnosis	/	1.00 [1.00, 4.00]^b^	1.00 [1.00, 4.00]^b^	3.00 [1.00, 5.00]^a^	< 0.001
Number of MCI diagnosis	/	2.00 [1.00, 4.00]^b^	3.00 [2.00, 5.00]^a^	1.00 [1.00, 1.00]^c^	< 0.001

P value indicates significance using ANOVA for normally distributed continuous variables, Kruskal–Wallis test for ordinal variables, and Chi-squared test for categorical variables. Superscript letters denote significance at post-hoc analysis with a > b > c.

**Table 4:** Comparison of models assessing the predictive validity for the onset of dementia of consistent, reverter, and uncertain MCI diagnoses based on Conventional (Model 1), Neuropsychological (Model 2) criteria, and combined model (Model 3).

	Model 1	Model 2	Model 3
Conventional criteria			
Consistent MCI	3.00 (0.21)***		2.22 (0.26)**
Reverters	0.85 (0.27)		0.89 (0.29)
Uncertains	2.81 (0.22)***		1.97 (0.22)**
Neuropsychological criteria			
Consistent MCI		3.58(0.29)***	2.65 (0.32)**
Reverters		0.49 (0.42)	0.47 (0.43)
Uncertain		4.84 (0.30)***	4.04 (0.30)***
AIC	1781	1740	1725
R^2^	0.057	0.096	0.116
Num. events	148	148	148
Num. obs	958	958	958
PH test	0.031	0.093	0.014

Hazard ratio (SD), model goodness-of-fit indices (AIC and R^2^), number of events and observations, and proportional hazards test (PH test) are reported. The reference group is cognitively normal subjects.

***p < 0.001; **p < 0.01; *p < 0.05.

### Subjective memory complaints

Table 5 shows the comparisons of Cox models evaluating the predictive validity for dementia onset according to the relative presence of subjective and objective cognitive impairment at baseline. SMI (subjective memory impairment, with normal cognition) at baseline was associated with a slightly increased risk of incident dementia only within the Conventional criteria framework. The CIND category (Cognitive Impairment no Dementia, characterized by objective impairment without subjective complaints) was associated with an increased risk of dementia when both the Conventional and modified Neuropsychological criteria (considering the presence of subjective cognitive complaints to diagnose MCI) were adopted, similar to MCI diagnosis based on Neuropsychological criteria. Interestingly, when the advanced classification was used, the highest predictive validity for dementia diagnosis was found when both subjective cognitive complaints and objective impairment were present (MCI diagnosis on the basis of Conventional and modified Neuropsychological criteria).

**Table 5:** Comparison of models assessing the predictive validity for the onset of dementia of the advanced classification based on conventional (Model 1), neuropsychological (Model 2) and modified neuropsychological criteria (Model 3).

	Model 1	Model 2	Model 3
Conventional criteria			
SMI	1.52 (0.29)		
CIND	2.31 (0.25)***		
MCI	4.47 (0.25)***		
Neuropsychological criteria			
SMI		1.21 (0.29)	
MCI		2.93 (0.22)***	
Neuropsychological criteria modified			
SMI			1.21 (0.29)
CIND			2.05 (0.24)**
MCI			4.40 (0.23)***
AIC	1738	1745	1733
R^2^	0.056	0.047	0.060
Num. events	145	145	145
Num. obs	936	936	936
PH test	0.178	0.041	0.094

Hazard ratio (SD), model goodness-of-fit indices (AIC and R^2^), number of events and observations, and proportional hazards test (PH test) are reported. The reference group is cognitively normal subjects.

***p < 0.001; **p < 0.01; *p < 0.05.

## Discussion

Early and informative MCI diagnosis is crucial for planning timely and appropriate interventions aimed at dementia risk reduction. However, to date, there is great heterogeneity in the methods and instruments used to formulate MCI diagnoses in clinical settings, and few studies have been performed to directly compare the prognostic value of different diagnostic algorithms. The primary aim of the present study was to compare the prognostic value of Conventional and Neuropsychological criteria for MCI diagnosis in a population-based sample of older adults who were followed for 12 years and resided in Italy. In particular, our research compared the ability of each classification method to detect subjects who then progressed to dementia within the study period and to verify the consistency of MCI diagnosis over time. Moreover, we further explored the predictive value of subjective memory complaints, with and without objective impairment, on clinical progression within each diagnostic framework.

In our research, which was conducted in a nonclinical setting, we found that the criteria used for the MCI diagnosis had a great impact on the number of cases detected, with many more MCI diagnoses performed using the Neuropsychological criteria (43.8% vs. 18.5%). Moreover, a baseline MCI diagnosis was associated with a twofold increased risk of developing dementia over 12 years, regardless of the criteria used to perform the diagnosis. Additionally, diagnoses performed with the Neuropsychological criteria were more consistent across the observation period than those performed according to the Conventional criteria (63.2% vs. 43.2%). Furthermore, even for those with uncertain diagnostic consistency due to incomplete assessments, an initial MCI diagnosis based on Neuropsychological criteria conferred the highest increased risk for dementia.

Another important and original finding is that the presence of subjective cognitive complaints in those with MCI was associated with the highest risk of developing dementia within both the Conventional and the Neuropsychological criteria frameworks.

The finding of a higher frequency of MCI cases detected via the Neuropsychological criteria contrasted with the findings of previous studies, which revealed that the Conventional criteria tended to detect more cases and presented higher false-positive rates^6,7,16,17,19^. This discrepancy could be explained by the specific features of our sample, which was a population-based cohort of individuals in a specific age range at baseline^24^ (70–75 years). Thus, the prevalence of subjective cognitive complaints in our sample was probably lower than that in the clinical setting, leading to a lower probability of being diagnosed with MCI on the basis of Conventional criteria.

Our results are in line with those of a previous study that revealed a comparable risk of dementia in individuals with a baseline MCI diagnosis according to the Neuropsychological criteria compared to Conventional ones^6,16,19^. In contrast, a previous study revealed a better ability of the Neuropsychological criteria to identify people who will progress to dementia^16^. The Neuropsychological criteria have been shown to be more effective at identifying individuals who will progress to dementia even in a sample of oldest old people^6^. The discrepancies found could be due to heterogeneity in the study setting and characteristics of the samples, since previous studies were conducted on longitudinal cohorts of community-dwelling older adults mostly displaying high levels of education^6,16,19^, whereas our sample is highly heterogeneous in terms of educational level (see Table 6 for details) and is thus more representative of the aged population.

**Table 6:** Neuropsychological tests means and standard deviations of the InveCe.Ab normative sample stratified for educational attainment and age range.

Age range	Levels of education	70–77 years			78–88 years		
		N	Mean	SD	N	Mean	SD
Rey auditory-verbal learning test, immediate recall	Less than primary school	74	33.63	7.03	18	32.60	7.96
	Primary school	368	35.28	8.73	113	35.52	8.10
	Middle school	245	37.32	8.93	88	38.00	8.17
	High school or higher	79	40.05	8.56	34	41.67	8.04
Rey auditory-verbal learning test, delayed recall	Less than primary school	74	6.69	2.52	18	7.30	2.11
	Primary school	368	6.85	2.72	113	7.42	2.62
	Middle school	245	7.04	2.78	88	8.00	2.74
	High school or higher	79	7.46	3.33	34	7.87	2.56
Rey-Osterrieth complex figure copy	Less than primary school	74	25.00	5.94	18	28.50	3.54
	Primary school	368	27.96	4.75	113	27.34	4.35
	Middle school	245	30.07	4.57	88	29.37	3.47
	High school or higher	79	31.76	3.46	34	32.00	2.18
Rey-Osterrieth complex figure recall	Less than primary school	74	12.82	4.08	18	12.00	0.00
	Primary school	368	12.12	4.80	113	12.08	4.40
	Middle school	245	13.79	5.12	88	13.32	5.75
	High school or higher	79	15.55	5.69	34	16.22	6.13
Clock drawing test	Less than primary school	74	18.11	2.22	18	16.59	2.15
	Primary school	368	18.90	1.38	113	18.32	1.29
	Middle school	245	19.21	1.35	88	18.72	1.04
	High school or higher	79	19.37	0.92	34	18.47	1.58
Trial making test A	Less than primary school	74	69.08	24.46	18	71.71	21.36
	Primary school	368	50.16	17.74	113	52.13	16.45
	Middle school	245	39.44	15.49	88	40.65	12.41
	High school or higher	79	36.23	11.02	34	40.50	16.48
Trial making test B	Less than primary school	74	149.00	38.05	18	197.00	57.24
	Primary school	368	139.66	59.33	113	169.39	67.58
	Middle school	245	95.09	39.38	88	117.15	49.38
	High school or higher	79	76.75	26.04	34	102.06	43.18
Trial making test B-A	Less than primary school	74	88.00	36.38	18	147.00	69.09
	Primary school	368	90.80	52.22	113	117.44	62.54
	Middle school	245	56.07	33.41	88	76.50	45.17
	High school or higher	79	40.52	22.60	34	61.56	36.24
Raven’s coloured progressive matrices	Less than primary school	74	23.14	4.55	18	20.77	6.34
	Primary school	368	26.09	4.40	113	26.34	4.66
	Middle school	245	28.89	3.81	88	28.67	3.75
	High school or higher	79	30.95	3.73	34	30.50	4.27
Attentional matrices	Less than primary school	74	39.85	8.84	18	34.00	6.59
	Primary school	368	47.53	7.31	113	43.19	7.46
	Middle school	245	50.77	6.18	88	47.28	7.58
	High school or higher	79	53.17	5.72	34	47.678	6.90
Semantic verbal fluency test	Less than primary school	74	13.58	2.62	18	12.79	2.69
	Primary school	368	16.18	3.75	113	15.83	3.53
	Middle school	245	19.47	4.17	88	18.98	4.06
	High school or higher	79	23.20	4.13	34	21.24	4.81

Normative sample for 70-77 years was composed of individuals with stable normal cognition at baseline (2010) and first follow-up (2012). Normative sample for 78-88 years was composed of individuals with stable normal cognition at second (2014) and third follow-up (2018).

Previous research comparing the diagnostic stability of different MCI criteria has shown contrasting results. Overton and colleagues reported that Conventional and Neuropsychological criteria had similar reversion rates and diagnostic stability^5^. Nevertheless, other studies have shown that the diagnostic stability over time is greater when the Neuropsychological criteria are used^6,16^. In addition, Loewenstein and colleagues reported a higher reversion rate when the MCI diagnosis was based on impairment in a single test than when more tests were considered^18^. Our research, which assessed diagnostic consistency across a 12-year observation period, revealed increased consistency of MCI diagnosis with the Neuropsychological criteria (177 stable diagnoses, 63.2%) compared with the Conventional criteria (121 stable diagnoses, 43.2%). Nevertheless, those diagnosed with MCI consistently over the entire observation period showed an increased risk of dementia for both diagnostic frameworks. Furthermore, we showed that even a single MCI diagnosis based on Neuropsychological criteria (uncertain category) was highly predictive of dementia. This category, compared with the other categories, tends to be older at first diagnosis and displays faster progression to dementia, with a similar number of assessments (except for reverters; see Table 3). This could be because the Neuropsychological criteria are more sensitive than the Conventional criteria are.

Finally, when we separately considered the presence of subjective and objective impairment at baseline, we found that individuals with MCI with cognitive complaints (Conventional and modified Neuropsychological criteria) presented the highest risk for dementia. Moreover, the CIND category (objective impairment without subjective complaints) conferred a twofold greater risk of dementia than did cognitively normal individuals.

The inclusion of subjective complaints as a mandatory criterion to diagnose MCI has been questioned in recent studies, as patients tend to underestimate their difficulties as objective cognitive impairment emerges or worsens^20,21^. A previous study showed that memory concerns increased the risk of AD in individuals with MCI, but their predictive value decreased as memory function worsened^25^. The results of our research suggest that subjective memory complaints should be considered an indicator of a greater risk of progression to dementia in those with detectable cognitive impairment.

Our research has several limitations. Due to the long observation period, participation in the study decreased over the years. Furthermore, the generalizability of our results should be further tested in different settings since our cohort was composed of older adults residing in a specific geographic area. Moreover, since the present study was conducted on a population-based cohort, our findings could be applicable to individuals with similar sociodemographic characteristics from both clinical and nonclinical settings.

Despite these limitations, our study has several strengths. First, as previously mentioned, the population-based study design allowed us to reduce selection bias, thus offering a broad and heterogeneous picture of the population studied. In addition, we prospectively followed the participants for 12 years with periodic multidimensional assessments, which is a longer period than did previous research in the field^26^. Thus, considering the study design, which enrolled all individuals in a restricted age range at baseline, we followed our cohort throughout the aging process, from 70–75 years to over 80 years. Finally, we tested the relative contribution of subjective cognitive complaints and objective impairment to the risk of dementia, contributing to the literature debate about considering patient-reported complaints for MCI diagnosis.

### Conclusions and future directions

The present study showed that the Neuropsychological and Conventional criteria, applied to a general population of older adults in a nonclinical setting, displayed comparable prognostic values across a 12-year observation period. The fulfillment of the Neuropsychological criteria was more consistent across time, and diagnostic consistency was associated with increased dementia risk for both criteria. Moreover, we showed that the presence of subjective memory complaints in MCI individuals increased the risk of dementia regardless of the threshold set for cognitive impairment and could be considered an indicator of progression at early stages. Further studies are needed to directly test the prognostic value of different MCI diagnostic algorithms in other clinical and nonclinical settings. Our results pave the way for new studies to make MCI diagnosis more reliable and sensitive, thus contributing to improving dementia case finding and management.

## Methods

### Participants and procedures

The participants for the present study were recruited from InveCe.Ab study (Invecchiamento Cerebrale in Abbiategrasso, i.e., Brain aging in Abbiategrasso; ClinicalTrials.gov, NCT01345110), a population-based cohort of people born between 1935 and 1939 and living in the city of Abbiategrasso (Milan, Italy) on the prevalence day (November 1st, 2009). The aim of the InveCe.Ab study was to estimate the incidence of dementia and explore the sociodemographic, clinical, and lifestyle factors associated with aging and dementia^24^.

The enrolled participants (n = 1321) underwent periodic multidimensional assessment, comprising blood sampling, geriatric visits, neuropsychological assessments, and social and lifestyle interviews (at baseline and after 2, 4, 8 and 12 years).

For the aim of the present study, we included individuals without a baseline diagnosis of dementia or major psychiatric disorders (depression or psychosis) and who performed at least one follow-up visit across the study period. Furthermore, among eligible individuals, at each assessment wave, we analyzed only patients completing at least 60% of the neuropsychological tests considered for the present study (7 out of 11). This pragmatic criterion was introduced to include only participants classified as cognitively normal or impaired based on a comprehensive evaluation of cognitive functions^23^.

All the participants provided written informed consent for the study procedures, which were in accordance with the Declaration of Helsinki. The study protocol was approved by the Ethics Committee of the University of Pavia on October 6, 2009 (Committee report 3/2009).

### Neuropsychological evaluation

All participants completed the same protocol of cognitive tests during a single session lasting 2 h. All tests were administered in Italian by neuropsychologists and are reported below:

- The geriatric depression scale short form^27^ (GDS-SF) is a useful screening tool to facilitate the assessment of depression in older adults. This is a brief, 15-item questionnaire in which participants are asked to respond by answering yes or no in reference to how they felt. Scores of 0–8 are considered normal; scores of 9–15 indicate depression.
- The mini-mental state examination^28^ (MMSE) is a screening test widely used to assess global cognitive function. It comprises a series of items to measure temporal and spatial orientation, immediate and delayed memory, language, attention, and visual construction. All correct items are summed to obtain a total score ranging from 0 to 30.
- The clock drawing test^29^ (CDT) is a fast and easily administered screening tool that covers a wide range of cognitive functions, including visuospatial and praxis abilities, selected and sustained attention, executive function and numerical knowledge. The participant is given a blank sheet of paper and asked first to draw the face of a clock, place the numbers on the clock, and then draw the hands to indicate 10 after 11. There are many different ways to score the clock-drawing test. We used a 20-point scale based on whether the sequence of numbers, the placement of numbers, and the placement of the hands were correct; each question was assigned one point if the drawing was correct and zero points if it was not correct.
- The Rey auditory verbal learning test^30^ (RAVLT) is a measure of a person’s ability to encode, combine, store and recover verbal information in different stages of immediate memory. It consists of five presentations of a 15-word list, each followed by attempted recall. This is followed by delayed recall after 15 min of nonverbal activities. The five recall trials were summed into one score (RAVLT Immediate), whereas delayed recall represented another score (RAVLT Delayed).
- Raven’s coloured progressive matrices^31^ (RCPM) is a nonverbal test typically used to measure abstract reasoning. The test includes 36 items, which are arranged into three sets (A, AB, B) of 12, listed in order of increasing difficulty. Each item is represented by a large square that contains a pattern with a piece missing. This requires the subject to select the missing piece among the six alternatives shown below. One point was given for each correct answer, and the total score was the sum of the correct answers (0-36).
- The attentional matrices^32^ is a test used to assess the ability of a participant to detect visual targets among distractors. It consists of crossing out as fast as possible target numbers of one, two or three digits in three matrices of numbers. The final score was the overall number of targets that were crossed out in 45 s.
- The trail making test^33^ (TMT) is a widely used test for assessing visual search, sustained and divided attention, set shifting and cognitive flexibility. It consists of two parts (A and B): In each part, the participant is asked to connect as quickly as possible a series of 24 consecutive circles that are randomly arranged on a page. TMT A uses all numbers, whereas TMT B contains both numbers and letters, requiring the patient to switch between them in consecutive order. The total score is the time needed to complete each part of the test in seconds, leading to three subscores: TMT A, TMT B and the subtraction between the two parts (TMT B-A).
- The Rey–Osterrieth complex figure^34^ (ROCF) is a commonly used neuropsychological assessment tool that assesses different functions, such as visuospatial abilities and memory and executive functions. It consists of the direct copying of a complex bidimensional figure and its recollection from memory after a 10-min delay. The maximum score for each task (direct copying and delayed reproduction) is 36: two points are given when the element is correctly reproduced; 1 point when the reproduction is distorted, incomplete but placed properly, or complete but in the wrong place; 0.5 points are given when the element is distorted or incomplete and in the wrong place; and 0 points are given when the element is absent or not recognizable.
- The semantic verbal fluency test^32^ is a short test frequently used in clinical and research practice to assess lexical retrieval and production. The participants are asked to generate as many words as possible falling into the categories “Colors”, “Animals”, “Fruits” and “Cities”, allowing 120 s for each category. According to the scoring procedures of the test version administered, the total score is the average of the correct words generated in the four categories.

To obtain individual Z scores to ascertain the presence of cognitive impairment according to Conventional and Neuropsychological criteria, we defined internal normative samples stratified by age and education. Specifically, a subsample of 766 participants with normal cognition (based on InveCe.Ab study criteria) both at baseline and after 2 years were used as normative samples for assessment waves 1–3 (age range of the normative sample: 70–75 years; age range of participants at waves 1–3: 70–77 years). An older normative sample for waves 4–5 was defined (age range of participants: 78–88 years), composed of 253 individuals displaying normal cognition both at waves 3 and 4 (age range: 78–83 years). Table 6 shows the means and standard deviations of the internal normative samples stratified for 4 levels of education (less than primary school, primary school, secondary school, high school or higher), used to compute individual Z scores.

### Group classifications

An algorithmic approach was applied retrospectively to the entire study sample to classify individuals according to the Conventional and Neuropsychological criteria. The presence of subjective cognitive complaints was based on the following yes/no question asked by the geriatrician: “Do you think you have some memory problems?”.

The presence of cognitive impairment was ascertained if at least one test was 1.5 SD below the normative mean for Conventional criteria and if two tests within the same cognitive domain were 1 SD below the normative mean for Neuropsychological criteria (except for the language domain where a single test was considered). Specifically, the following cognitive domains composed of one to three tests were defined: memory (RAVLT immediate and delayed scores; ROCF delayed recall); attention (Attentional Matrices, TMT A); executive functions (TMT B, TMT B-A, RCPM); visuospatial abilities (CDT, ROCF copy); and language (semantic fluency).

The absence of functional impairment was ascertained with the Basic Activities of Daily Living (BADL) and Instrumental Activities of Daily Living (IADL) scales^35,36^.

Using these measurements, we performed two different group classifications. The standard classification, which considers only the presence/absence of MCI, and the advanced classification, which also includes subjective memory impairment (SMI) as a separate diagnostic entity, includes individuals reporting subjective memory complaints to the geriatrician but displaying preserved cognitive functioning. Thus, advanced classification led to 4 subgroups for Conventional criteria: cognitively normal (CN), subjective memory impairment (SMI), MCI and cognitive impairment without memory complaints, which were defined as CIND (Cognitive Impairment No Dementia) in previous research^37^. The advanced classification with the Neuropsychological criteria, which does not require subjective cognitive complaints to make an MCI diagnosis, leads to 3 subgroups: CN, SMI and MCI. Furthermore, we also included a modified version of the Neuropsychological criteria leading to 4 subgroups: CN, SMI, MCI with cognitive complaints, and MCI without cognitive complaints (named CIND in accordance with the definition used for the Petersen criteria). This approach helps to assess the relative contribution of subjective cognitive complaints and objective cognitive impairment to clinical progression.

With respect to diagnostic consistency, participants with at least one MCI diagnosis across the observation period (12 years) were classified as consistent, reverter or uncertain MCI, following the Petersen and Neuropsychological criteria, respectively. We considered consistent MCI those individuals who, once diagnosed, keep fulfilling the criteria for cognitive impairment in subsequent waves (regardless of dementia diagnosis), in line with the ideal trajectory of MCI as a prodromal stage for dementia. We defined reverters as those who, after MCI diagnosis, were classified as cognitively normal in subsequent waves. Furthermore, we considered those with uncertain MCI those who could not be definitively classified as reverters or consistent due to incomplete or absent assessments after the first MCI diagnosis.

Those not fulfilling the criteria for cognitive impairment across the entire observation period were classified as cognitively normal.

### Outcome

The main outcome of the study was conversion to dementia within the observation period (12 years). Within the InveCe.Ab study, the diagnosis was reached through a two-step process: first, the neuropsychologist and the physician make independent working diagnoses; then, an expert geriatrician together with a clinical neuropsychologist reviewed all individual records to define the final diagnosis. Dementia diagnosis was formulated after multidimensional assessment at each evaluation wave according to the DSM-IV-TR^38^ (waves 1–3) or DSM-5 criteria^12^ (waves 4–5). If the diagnosis was first formulated after the study visits, dementia onset was set at the date of the corresponding evaluation wave, whereas if the diagnosis was formulated in clinical settings during the period between subsequent study visits, the date of diagnosis was retrospectively collected^39^.

### Statistical analysis

We preliminarily conducted a descriptive statistical analysis of the sociodemographic characteristics of the sample. To assess the agreement between the two MCI classifications, we subsequently employed Cohen’s kappa (κ), Jaccard’s index, and McNemar’s test.

To compare the operational characteristics of sensitivity and specificity for dementia detection between the two sets of criteria (standard binary classification), we performed an area comparison analysis under receiver operating characteristic (ROC) curves via the DeLong test. This analysis, together with the calculation of positive and negative predictive values (PPVs, NPVs), evaluated the ability of each MCI criterion to classify individuals who progressed to dementia over the following 12 years.

We subsequently developed Cox proportional hazards models adjusted for age, sex, and education to evaluate the predictive validity of each MCI criterion (standard classification), both separately and combined, for the onset of dementia over time. Comparative analysis of the models was performed using the Akaike information criterion (AIC), R-squared values, and hazard ratios.

Agreement in diagnostic consistency between the two classifications was analyzed via Cohen’s kappa, Jaccard’s index, and McNemar’s test, with a focus on individuals who were diagnosed with MCI at least once under both criteria. Additional descriptive statistics and Cox models adjusted for age, sex, and education were developed to assess the impact of consistency and uncertainty in MCI diagnosis on the prediction of dementia over time. This comparison was based on the evaluation of the AIC, R-squared values, and hazard ratios.

Finally, we performed Cox proportional hazards models adjusted for age, sex, and education to compare the predictive validity for dementia onset over time, according to advanced classifications (CN, SMI, MCI, CIND) based on Petersen, Neuropsychological, and Neuropsychological modified criteria.

## Supplementary Information

Supplementary Information.

## Data Availability

The data that support the findings of this study are available on reasonable request from the corresponding author.
