# Correction: Conventional and neuropsychological criteria for mild cognitive impairment show similar prognostic value for dementia across 12 years in a non-clinical setting

**DOI:** 10.1038/s41598-025-11549-y

**Published:** 2025-07-22

**Authors:** Simone Galati, Michele Rossi, Federica Del Signore, Giulia Locatelli, Antonio Guaita, Elena Rolandi

**Affiliations:** 1 Golgi Cenci Foundation, Corso San Martino 10, 20081 Abbiategrasso (MI), Italy; 2 Department of Brain and Behavioral Sciences, University of Pavia, Pavia, Italy; 3 Department of Psychology, University of Milan-Bicocca, Milan, Italy

Correction to: *Scientific Reports* 10.1038/s41598-025-04275-y, published online 05 June 2025

The original version of this Article contained errors in the name of authors Simone Galati, Michele Rossi, Federica Del Signore, Giulia Locatelli, Antonio Guaita & Elena Rolandi, which were incorrectly given as Galati Simone, Rossi Michele, Del Signore Federica, Locatelli Giulia, Guaita Antonio & Rolandi Elena respectively.

The original Article has been corrected.

