# Supplementary material for: Conventional and neuropsychological criteria for mild cognitive impairment show similar prognostic value for dementia across 12 years in a non-clinical setting
Source: Sci Rep. 2025 Jun 5;15:19827. doi: 10.1038/s41598-025-04275-y (PMC12141691; doi:10.1038/s41598-025-04275-y)
Supplement: Supplementary file 1 — Supplementary Information. [file 41598_2025_4275_MOESM1_ESM.docx]

**Conventional and Neuropsychological criteria for Mild Cognitive Impairment show similar prognostic value for dementia diagnosis across 12 years in a nonclinical setting**

Galati Simone, Rossi Michele, Del Signore Federica, Locatelli Giulia, Guaita Antonio, Rolandi Elena

**Supplementary Table S1:** Diagnostic outcomes (advanced classification) at each timepoint based on Conventional Criteria

|  | **Conventional Criteria** | | | | |
| --- | --- | --- | --- | --- | --- |
|  | **Baseline**  **n=936** | **2-years follow-up**  **n=842** | **4-years follow-up**  **n=725** | **8-years follow-up**  **n=544** | **12-years follow-up**  **n=337** |
| **CN** | 320 (34%) | 266 (31%) | 197 (27%) | 124 (23%) | 59 (17%) |
| **SMI** | 177 (19%) | 154 (18%) | 114 (16%) | 123 (23%) | 40 (12%) |
| **MCI** | 173 (19%) | 192 (23%) | 195 (27%) | 140 (26%) | 120 (36%) |
| **CIND** | 266 (28%) | 200 (24%) | 204 (28%) | 139 (25%) | 96 (28%) |
| **Dementia** | 0 | 5 (1%) | 14 (2%) | 17 (3%) | 21 (6%) |
| **Missing** | 0 | 25 (3%) | 1 (0%) | 1 (0%) | 1 (0%) |

Abbreviations: Cognitively Normal (CN), Subjective Memory Impairment (SMI), Mild Cognitive Impairment (MCI), Cognitive Impairment Not Dementia (CIND).

**Supplementary Table S2:** Diagnostic outcomes (advanced classification) at each timepoint based on Neuropsychological criteria

|  | **Neuropsychological Criteria** | | | | |
| --- | --- | --- | --- | --- | --- |
|  | **Baseline**  **n=936** | **2-years follow-up**  **n=842** | **4-years follow-up**  **n=725** | **8-years follow-up**  **n=544** | **12-years follow-up**  **n=337** |
| **CN** | 344 (37%) | 291 (34%) | 229 (32%) | 149 (27%) | 71 (21%) |
| **SMI** | 182 (19%) | 161 (19%) | 129 (18%) | 139 (26%) | 54 (16%) |
| **MCI** | 410 (44%) | 371 (44%) | 352 (48%) | 238 (44%) | 190 (57%) |
| **Dementia** | 0 | 5 (1%) | 14 (2%) | 17 (3%) | 21 (6%) |
| **Missing** | 0 | 14 (2%) | 1 (0%) | 1 (0%) | 1 (0%) |

Abbreviations: Cognitively Normal (CN), Subjective Memory Impairment (SMI), Mild Cognitive Impairment (MCI).
